# Effect of in-hospital comprehensive geriatric assessment (CGA) in older people with hip fracture. The protocol of the Trondheim Hip Fracture Trial

**DOI:** 10.1186/1471-2318-11-18

**Published:** 2011-04-21

**Authors:** Olav Sletvold, Jorunn L Helbostad, Pernille Thingstad, Kristin Taraldsen, Anders Prestmo, Sarah E Lamb, Arild Aamodt, Roar Johnsen, Jon Magnussen, Ingvild Saltvedt

**Affiliations:** 1 Department of Geriatrics, St. Olav Hospital, University Hospital of Trondheim, Norway; 2 Department of Orthopaedic Surgery, St. Olav Hospital, University Hospital of Trondheim, Norway; 3 Department of Neuroscience, Norwegian University of Science and Technology, (NTNU), Trondheim, Norway; 4 Clinical Trials Unit, University of Warwick, UK; 5 Department of Public Health and General Practice, Norwegian University of Science and Technology (NTNU), Trondheim, Norway

## Abstract

**Background:**

Hip fractures in older people are associated with high morbidity, mortality, disability and reduction in quality of life. Traditionally people with hip fracture are cared for in orthopaedic departments without additional geriatric assessment. However, studies of postoperative rehabilitation indicate improved efficiency of multidisciplinary geriatric rehabilitation as compared to traditional care. This randomized controlled trial (RCT) aims to investigate whether an additional comprehensive geriatric assessment of hip fracture patients in a special orthogeriatric unit during the acute in-hospital phase may improve outcomes as compared to treatment as usual in an orthopaedic unit.

**Methods/design:**

The intervention of interest, a comprehensive geriatric assessment is compared with traditional care in an orthopaedic ward. The study includes 401 home-dwelling older persons >70 years of age, previously able to walk 10 meters and now treated for hip fracture at St. Olav Hospital, Trondheim, Norway. The participants are enrolled and randomised during the stay in the Emergency Department. Primary outcome measure is mobility measured by the Short Physical Performance Battery (SPPB) at 4 months after surgery. Secondary outcomes measured at 1, 4 and 12 months postoperatively are place of residence, activities of daily living, balance and gait, falls and fear of falling, quality of life and depressive symptoms, as well as use of health care resources and survival.

**Discussion:**

We believe that the design of the study, the randomisation procedure and outcome measurements will be of sufficient strength and quality to evaluate the impact of comprehensive geriatric assessment on mobility and other relevant outcomes in hip fracture patients.

**Trials registration:**

ClinicalTrials.gov, NCT00667914

## Background

Every year about 9000 persons undergo hip fracture surgery in Norway [1]. Hip fractures among older people are associated with high morbidity, mortality, disability and subsequent hospital and societal costs as well as reduction in quality of life [2-6]. A Study from Oslo, Norway showed that the proportion of patients living in nursing homes increased from 15% before to 30% after the hip fracture; the proportion walking without any aid decreased from 76 to 36%; and 43% of the patients lost their pre-fracture ability to mobilise outside their own home [7].

Older people with hip fracture often have extensive co-morbidity which is associated with functional impairments and frailty. The frailty phenotype is defined by deterioration of multiple organ systems including the neurological, musculoskeletal, cardiovascular, metabolic or immunological systems [8]. Frailty has been shown to be associated with falls resulting in injuries [9].

Previous studies show improved outcomes when older people with hip fracture are cared for by a specialist multidisciplinary team [10-12]. Reports indicate improved efficiency of multidisciplinary geriatric rehabilitation especially regarding delirium, recurrent falls and fractures, and use of institutional care [13-15]. There is now a growing body of evidence supporting this approach [16,17] and recently evidence-based guidelines as for treating hip fracture patients have been developed,[18] although context and organisation of so-called hip-units differs widely [19].

However, the findings in these studies are not conclusive and we still do not know which specific input, if any, is crucial to beneficial effects. Is it the management of medical complications; is it a goal-oriented intervention by one single professional staff-member, i.e. the physiotherapist, nurse or physician; or is it related to a multi-component mix of some or all these?

In a previous study we have shown that treating acutely sick and frail older patients in a care pathway based on a geriatric evaluation and management service significantly reduced mortality and also improved patients' chances of living at home [20,21]. Therefore, it would seem reasonable that frail old hip fracture patients would benefit from comprehensive geriatric assessment (CGA) in the acute setting. Evaluation of efficiency of care pathways for hip fracture patients should emphasise both survival, general function; especially mobility and physical activity, but also quality of life (QoL) and caregiver burden, as well as costs. There is a strong focus in health care management today on the efficient use of limited resources, especially on shortening of length of stay (LOS) and lowering of costs. Furthermore, over the years LOS for patients with hip fracture has declined irrespective of settings and the organizing of health care, indicating that new models of care are less costly than traditional clinical pathways. However, shortening of LOS and reduced emphasis on acute and in-hospital rehabilitation may increase admission rates to nursing homes and reduce the quantity and quality of rehabilitation, and consequently reduce recovery of walking ability and function [22,23] and also shift costs between sectors.

In the present study we aim to investigate whether an alternative clinical pathway for hip fracture patients during the in-hospital acute phase applying CGA in an orthogeriatric ward may improve outcomes in the short (1 and 4 months postoperatively) and long (12 months postoperatively) term without introducing additional specific follow-up programs. Hopefully we will increase the knowledge of whether in-hospital treatment of hip fracture patients in a geriatric acute-unit primarily will improve mobility, and secondly increase the chance of being discharged to and live in their own homes, and improve function and self-rated health, while maintaining the new care pathway cost-neutral in comparison to treatment in a traditional orthopaedic unit.

In accordance with general guidelines for the development, evaluation and reporting of randomized controlled trials (RCT) for complex interventions [24] the purpose of the this paper is to present context and study design, a short description of intervention, outcome measures and power calculations and also procedures for the Trondheim Hip Fracture Trial. An extensive report on the intervention program will be published later.

### Aims

#### Primary aim

• To estimate the effect on mobility 4 months after surgery of treating hip fracture patients in an orthogeriatric ward as compared to treatment in an orthopaedic ward.

#### Secondary aims

• To estimate the effect of the intervention on place of residence, gait, activities of daily living, mood and health related quality of life 1, 4 and 12 months postoperatively.

• To investigate change in gait control and daily physical activity through one year after surgery.

• To estimate the effect of the intervention on the use of health care resources and survival.

• To estimate the effect of the intervention on fear of falling and falls 4 and 12 months postoperatively.

## Methods/design

### Project context

The present study is conducted at St. Olav Hospital, the University Hospital of Trondheim, Mid-Norway. St. Olav Hospital also serves as a local hospital for 280.000 inhabitants of Soer-Troendelag County, admitting all hip fracture patients from this catchment area.

During the study period the Department of Orthopaedics will run a Trauma Unit consisting of 19 beds for inpatient orthopaedic care. While in the Emergency Department hip fracture patients are examined by the orthopaedic resident on call who in collaboration with the orthopaedic surgeon in charge establishes diagnoses and indication for surgery.

The Department of Geriatrics is organised as a formal unit of the Clinic of Internal Medicine consisting of a 10 bed-ward of acute geriatrics services linked to an out-patient facility. During a recent hospital reorganisation with cutting down of beds in the Department of Orthopaedics an orthogeriatric 5 bed-unit was established as an additional but still integrated part of the acute geriatric ward.

Being a new service for hip fracture patients routinely offered in parallel with the traditional orthopaedic care pathway, it was decided to evaluate potential benefits of this unit, now investigated through the present study. Enrolment of study patients was planned to start after a 4 months clinical run-in period for the new unit.

### Study design

The study is designed as a RCT with parallel groups where the intervention of interest, a CGA and management of hip fracture patients taking place in this orthogeriatric unit is compared with traditional care in an orthopaedic ward.

### Study population

All people over 70 years of age, with an acute hip fracture, previously being able to walk 10 meters, and living in their own homes or staying temporarily in an institution, suffering an intracapsular, trochanteric or subtrochanteric fracture, and able to give an informed consent, are invited.

Excluded are patients with pathological fractures or multi trauma injuries or with terminal illness not expected to live longer than 3 months or patients who have already been enrolled in this study. At study start the catchment area consisted of the City of Trondheim and the nearest municipalities. In case of slow recruitment we will use the option of expanding the catchment area to comprise all municipalities of Soer-Troendelag.

### Intervention

Patients randomised to the intervention group are transferred directly from the Emergency Department to the orthogeriatric ward while control patients are transferred to the trauma unit at Department of Orthopaedic Surgery. Orthopaedic surgeons are responsible for the initial assessment, diagnosing of the fracture and decisions on type of surgery for both groups. Anaesthesiologists make preoperative assessments regarding analgesia, operability and perioperative anaesthesiological procedures. After surgery and for a limited time period all patients are observed in the recovery unit.

On request orthopaedic surgeons examine study patients in the orthogeriatric ward and supervise the staff. Geriatricians serve the orthopaedic ward equivalently.

The experimental intervention program is offered only during the acute hospital stay. The orthopaedic surgeons decide on traditional follow-up consultations after discharge irrespective of group allocation.

#### Experimental group

Physicians at the Department of Geriatrics or residents on call have the 24-hour medical responsibility pre- and postoperatively.

The treatment strategy is based upon CGA which is a systematic and multidimensional diagnostic process focusing on evaluation of frail elderly persons' medical, psychosocial and functional capabilities and limitations in order to develop a coordinated and integrated plan for treatment and long-term follow-up by the primary health care system [25]. An interdisciplinary team consisting of geriatricians and residents, nurses, physiotherapists and occupational therapists with special competence in geriatrics is responsible for the CGA program. The team emphasizes adequate nutrition, early mobilization and functioning in activities of daily living, initial in-hospital rehabilitation and early discharge planning. Discharge planning starts as early as possible involving all team members. Whenever possible, patients are recommended to receive post discharge rehabilitation in their own home. In addition to treatment of current medical conditions, the management program also focuses on factors related to the fall incident causing the fracture.

#### Control group

Control patients receive traditional treatment at the Trauma Unit and follow-up at the Orthopaedic Out-patient Clinic. All patients are referred for in-hospital physiotherapy. Staff nurses are responsible for the discharge planning.

### Measures

***Mobility*** as primary outcome is assessed using the composite measure of the Short Physical Performance Battery (SPPB) [26,27]. SPPB consists of three tasks: 10 second of standing balance in three different positions (side-by-side, semi-tandem and tandem); 4 meter timed walking at preferred speed; and time to rise from a chair five times [26]. Each task is scored on a 0-4 scale. A score of 0 is given if the participant is unable to complete the task. Scoring from 1-4 for each task is assigned based on quartiles of performance derived from the Established Populations for the Epidemiologic Study of the Elderly (EPESE) [27]. A summary score ranging from 0-12, with 12 as the best score is created by summation of scores from the three tasks. The test is suitable for scoring persons with a large range of functional levels. It has been shown to have acceptable internal consistency (Chronbach alpha = 0.76) and test-retest reliability [28], ability to predict functional decline, rehospitalisation and death in older patients after hospitalization [29] and also to measure change in mobility in hip fracture patients.

***Mobility ***as secondary outcome is measured by the Timed Up & Go (TUG). According to the procedure time needed to rise from a chair, walk 3 meters, turn and walk back and sit down is measured [30]. The test is performed twice and the mean time (seconds) of the two trials is used as outcome. In the original paper by Podsiadlo the second of two trials is used, while in an earlier intervention study we have described high reliability of using the mean of two [31]. For participants not able to complete two trials, only one trial is used. Participants are instructed to use walking aids support if used regularly. Repeated tests aim to obtain fast speed while preserving safety, irrespective of using walking aids or not. TUG is well validated [30] and has been used in several studies on hip-fracture patients to predict falls [32], to assess functional mobility [33-35] and to assess effect of home-based therapy [36]. A limitation of using TUG is that scoring presupposes that the person is able to perform all sub-components of the task. Mobility and mobilisation during the index stay will be measures by use of Cumulated Ambulation Score (CAS) [37].

***Place of residence*** is used as a secondary outcome. Registrations of place of residence and change in place of residence are based on Gerica - the Electronic Health Record (EHR) of municipality of Trondheim by a procedure similar to one we have reported previously [20]. The typology differentiates between patients living in their own home, sheltered housing, nursing home, rehabilitation facility or hospital, respectively.

***Activities of daily living (ADL) ***is measured using the Barthel Index [38] and Nottingham extended I-ADL scale [39] based on reports, if possible from the patient, from next of kin or from nursing staff. Supplementing Gerica ADL-scores are filled in by community nursing staff. The Barthel Index evaluates a patient's self-care abilities in 10 areas, including bowel and bladder control. The scoring depends on the person's need for help such as in feeding, bathing, dressing, and walking. The Barthel index was constructed for stroke patients but has also been extensively used in hip fracture patients. I-ADL scales measure a series of life functions necessary for maintaining a person's immediate environment-eg, obtaining food, cooking, laundering, house cleaning and phone use. The Nottingham extended I-ADL scale has been shown to be reliable and valid in patients undergoing surgery for osteoarthritis but may underestimate the sizes of the health gain, at least after arthoplasty [40].

#### Health Related Quality of Life

The EuroQol is a widely-used standardised measure of self reported health [41] using questions in five domains (EQ-5D) that is applicable to a wide range of health conditions and treatments providing a simple descriptive profile and a single index value for health. ***Pain*** is measured by a numeric rating scale (NRS) (0-10) [42]. The Charnley's Hip Score as used in the SAHFE protocol (Standardized Audit of Hip Fractures in Europe) is used as a supplement [43].

#### Gait

Gait assessments are recorded for a subset of participants being able to walk without assistance from another person and attending the 4- and/or 12-month evaluations at the outpatient clinic. These measurements are performed using an electronic gait mat GaitRite^® ^which is regarded a reliable measure of spatio-temporal gait parameters also in elderly and frail people [44-46]. Participants should preferably walk the gait mat without walking aids. ***Physical activity ***is monitored in all patients when sensors are available for use. For these measurements we are using the small body worn accelerometer-based sensor ActivPal^®^[47], which is undergoing extensive evaluation in our research group [48].

#### Falls and fear of falling

Number of falls and fall related injuries are registered retrospectively in three ways at each follow-up; through medical records, and asking the patient and the next of kin. Fear of falling is assessed by a) asking a simple question: "Are you afraid of falling"-yes/no scored on a simple four-point Likert scale [49] and b) by applying the 7-item Falls Efficacy Scale International (FES-I) [50].

***Cognitive function ***is measured by use of the Clinical Dementia Rating scale (CDR)[51] based on registrations from next of kin and the performance based screening tool of patients, the Mini Mental State Examinations (MMSE)[52].

#### Depression

To assess the effect of the intervention on depressive symptoms we use the Geriatric Depression Scale 15 (GDS-15) [53-55]. GDS-15 can be interpreted as an indication of presence/absence of depressive mood [56,57].

#### Health economics

We will compare direct costs related to treatment in the orthogeriatric ward vs orthopedic ward, readmissions, rehabilitation, care in institutions, and home care services by calculating the incremental cost effectiveness ratio (ICER) and use a non-parametric bootstrapping approach. We will assign a value to the EuroQol states using previously developed tariffs of values. Robustness of results to choice of value set will be discussed. Where there are incomplete (censored) benefits or cost data due to loss to follow-up we will use non-parametric methods to infer cumulative costs and benefits [58,59]. Information on time of death will be collected from the National Registry.

#### Hospital related information

Data on cause and duration of any hospital admissions during the trial period is extracted from participants' hospital records. Hospital records will also be the most important information source for medication, previous and present co-morbidity and data related to pre-, peri- and postoperative monitoring.

#### Consent and enrolment

Nurses on call in the Emergency Department will undertake eligibility screening of all hip fracture patients. If there is a free bed in the orthogeriatric unit patients fulfilling the inclusion criteria are informed about the study and asked to participate. Depending on general health, pain, anxiety and fatigue study information is given as a short version. Proxies are informed about the study when appropriate and/or available, especially in relation to patients whose consenting competence could be questioned. Written consent is collected primarily at admittance in the Emergency Department or occasionally on day 3 or 5 at the clinical ward where research assistants routinely give a second orally, and also a written version of the study information to be kept by the patient or proxy. Furthermore, participating patients consent to participation for all four data collection points, otherwise being excluded. Explicit oral consent is accepted for patients unable of writing. At each data collection point participants receive repeated information on the study.

#### Randomisation and allocation concealment

After giving their informed consent participants are enrolled and randomized to immediate transfer for medical treatment in the orthogeriatric unit followed by surgical treatment by orthopaedics and further geriatric work-up and management in the orthogeriatric unit, or to receive traditional care in the Department of Orthopaedics. Randomisation is performed by using a web-based computerised randomisation service at the Unit of Applied Clinical Research, NTNU. Randomisation is blocked, with a random block length being integrated into the programming.

Research assistants are monitoring all hip fracture patients admitted to the hospital. Occasionally eligible patients may mistakenly be transferred to the Orthopaedic Department without being evaluated for eligibility. If not already being transferred for immediate surgical treatment and within 24 hours since admittance, patients are informed about the study and asked to participate. If patients consent, they are enrolled and randomised according to the protocol. After surgical treatment these patients are transferred to the orthogeriatric unit or returning to the orthopaedic unit according to results of the randomisation.

#### Data collection

For practical reasons it is not possible to implement systematic blinding of testing during the hospital stay. For the 1-, 4-, and 12-month assessments testers will not have access to information about the patients' group assignment.

Background information on living conditions, physical and cognitive function before the fracture is collected for all participants starting already during the stay in the Emergency Department. On day 3 or 5 research assistants collect details from patients' on falls history, use of mobility aids, pre-fracture scoring of Barthel ADL-Index and Nottingham extended I-ADL Scale when the clinical condition makes it appropriate, or from proxies when they are available. These registrations will be used as explanatory variables in the statistical analyses.

Mobilisation is monitored using CAS during the 3 first days after the operation. On day 3 a research assistant attaches an ActivPal sensor anteriorly on the non-affected thigh for at least a 24-hour activity monitoring. The sensor is removed on day 5. On day 5 or the nearest working day a SPPB mobility score is completed by a research assistant.

Research assistants will continually scrutinise study forms on missing data. Missing data from proxies are collected through telephone calls, as is also information needed to fill in the CDR form. Electronic hospital records will give further information on clinical examinations, medication, blood tests and other investigations performed during the index stay.

The 1-month registration is performed by research assistants at the site where the patient is living, irrespective of location. This might be the patient's own home, a nursing facility or a rehabilitation institution. The time window is 4 weeks ± 5 days. For details on data collection and questionnaires, see Table 1. Information on Barthel ADL Index or Nottingham extended I-ADL Scale items are collected primarily from the patients depending of cognitive function, or alternatively from the proxy. Whenever possible, information on participants living at remote locations from St. Olav Hospital is collected by trained local physiotherapists hired as research assistants.

**Table 1:** Measures, scales, questionnaires and time-points of data collection

Index stay	1 month postoperatively	4 and 12 months postoperatively
CAS (3 days)		
SPPB	SPPB	SPPB
TUG	TUG	TUG
	Place of residence	Place of residence
	MMSE	MMSE
	GDS-15	GDS-15
	FES-I	FES-I
	EQ-5D	EQ-5D
	NRS-pain	NRS-pain
ActivPal (24 hours)		ActivPal (4 days)
		GaitRite
Hand grip strength	Hand grip strength	Hand grip strength
		Quadriceps strength
Before fracture:		
Barthel Index	Barthel Index	Barthel Index
NEIADL	NEIADL	NEIADL
Falls	Falls	Falls
Walking aids	Walking aids	Walking aids
CDR	CDR	CDR

Abbreviations: CAS = Cumulative Ambulation score, CDR = Clinical Dementia Rating, EQ-5D = EuroQual-5 Domains, FES-I = Falls Efficacy Scale-International, GDS-15 = Geriatric Depression Scale, MMSE = Mini Mental State Examination, NEIADL = Nottingham extended Instrumental Activities of Daily Living, NRS = Numeric Rating Scale, SPPB = Short Physical Performance Battery, TUG = Timed Up & Go.

The 4-month registration is performed by a research assistant at the hospital out-patient facility applying the present infrastructure for testing aspects of mobility and gait using the electronic gait mat. To secure maximal study compliance and low attrition rate, transportation both to and fro is taken care of by the same experienced taxi driver. An ActivPal sensor is worn for at least a 96-hour period of activity monitoring. Participants are urged to be tested at the hospital. In case of extensively impaired physical or mental capacity a pragmatic and reduced test protocol is applied in their own home or where they are staying for the time being. The time window is 4 months ± 2 weeks. For details on data collection and questionnaires, see Table 1.

The 12-month registration is performed similar to the 4-month registration. The time window is 12 months ± 4 weeks. For details on data collection and questionnaires, see Table 1.

#### Adverse event management

Mortality rate is closely monitored. If the mortality rate becomes 50% higher for the intervention group the trial steering committee will be asked to evaluate individual case notes, reports and general aspects. The trial will be closed if the difference holds a significance level p < 0.10.

### Power and statistical analyses

Sample size estimates are based on mobility assessed by SPPB at 4 months following the fracture. A change in the SPPB score of 0.5 points is considered a small but meaningful change, while 1 point is considered a more substantial change. In order to detect an effect size of 1.0 when power is 80% and alpha = 0.05, a sample size of 304 participants would be needed. Based on data from a previous prospective observational study in a similar study population (work in progress), we expect a drop out rate of 10% due to death and 10% due to withdrawals during the first 4 months following the fracture. To allow for 304 patients to remain in the project at four months after the fracture 380 persons need to be included. Thus, the plan is to include a total sample of 400 participants. The assumptions underlying the sample size (i.e. the standard deviation at baseline) has been checked by an independent clinical trials unit after the first 200 patients enrolled, and found to be acceptable.

All data will be analysed and presented according to the updated CONSORT guidelines for reporting parallel group trials[60]. Patterns of missing data will be explored prior to analysis, and accounted for in the analysis by imputation methods [61]. To study differences in change between groups we will use multivariate analyses by use of mixed models for longitudinal data by general linear modelling (GLM) for continuous outcomes and by logistic regression for binary outcomes [62]. To study associations between the new clinical pathway and time to events, we will use Kaplan Meyer plots and the Cox proportional hazards regression model. In all analyses we will control for confounding factors and interactions and present both unadjusted and adjusted effects with 95% confidence intervals.

### Time plan of the study

**Figure 1:**
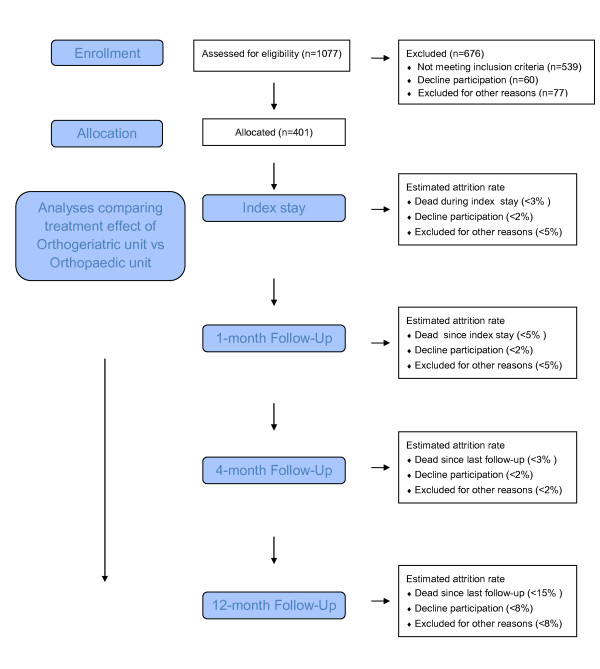
**Flowchart**. Overview of patients and attrition rates at enrollment, index stay and follow-up 1, 4 and 12 months after the hip fracture. Attrition rates at all assessment time points are presented as estimated percentages of participants allocated into the study (%). The estimates are based on preliminary data from an observational study in our department.

Since study start on April 18^th ^2008 until December 30^th ^2010 altogether 1077 hip fracture patients have been admitted to the Emergency Department at St. Olav Hospital, Trondheim University Hospital and screened for eligibility, of whom 401 have consented to participation, see Figure 1.

The final 12-month registrations will take place in December 2011. The formal analyses are estimated to start when the data base on the 4-month primary endpoint measures of mobility is finalised by May 2011. With exception of the study statistician, the study team will be masked from the trial results until the final follow up is completed.

#### Ethics and approvals

The study is approved by the Regional Committee of Ethics in Medical Research (Mid-Norway) (REK4.2008.335), the Norwegian Social Science Data Services (NSD19109), and the Norwegian Directorate of Health (08/5814).

## Discussion

Presenting this paper of the study protocol covering design, outcome measures, power calculations and procedures of the Trondheim Hip Fracture Trial is in accordance with general guidelines for reporting of RCT protocols for complex interventions [24], although it is published after the conclusion of the recruitment phase but still before the onset of data analysis and while the data collection is going on.

The objective of the study is to evaluate the impact of CGA on older hip fracture patients still having potential of functional improvement and preservation of health related quality of life aiming at prolonging their ability to live in their own home. Excluded are young hip fracture patients, patients with terminal illness, permanent nursing home residents and patients unable to walk. Patients with cognitive impairment and also temporary nursing home residents are included, representing patients known to be at especially high risk of further deterioration. Therefore, the study sample should comprise the most relevant segments of hip fracture patients regarding measurable benefits of CGA, being neither too healthy nor too ill.

We have chosen mobility as the primary endpoint, mainly because impaired mobility is one of the most feared consequences of a hip fracture in addition to death and nursing home placement, hopefully being accessible for intervention [16]. Still, potential benefits of CGA on mobility at 4 months will be more or less an indirect consequence of the intervention. Although mobility has been defined as the most important outcome, several secondary study outcomes i.e. ADL and I-ADL, health related quality of life, the extent of being discharged to own home, and costs may be equally relevant. This study is not sampled for mortality and nursing home placement as endpoint, and thus this information will only be used for hypothesis generation for future studies.

The context and organisation of care pathways for hip fracture patients differ extensively even in a small country like Norway. Nevertheless, there are consistent efforts by hospital managements towards shortening of hospital LOS based on fast-track orthopaedic services. Important consequences are less time for stabilisation of clinical conditions, assessment and treatment of relevant co-morbidity, as well as shifting of rehabilitation services out of hospitals, contrasting important constitutive elements of CGA-based specialist services.

Since the present intervention program will not implement any kind of medically follow-up by geriatric specialist services, and recommendations are to be dealt with by general practitioners and nursing homes or rehabilitation facilities outside hospital, important aspects of CGA may be lost. However, the competence and compliance of primary health care system vary extensively. Limitations of the study might thus be related both to study sample, non-blinding of assessors and choice of endpoints, as well as content and performance of the experimental intervention program.

The most important challenge is still the black box of inter-linked elements of CGA, of which we still do not know what is actually working. Therefore, evaluating the benefit of CGA within the present context without including an extended and optimal geriatric rehabilitation service or a relevant follow-up program after discharge from hospital may in fact increase the knowledge base as to the most important elements of CGA. The present study will hopefully be able to designate potential predictors of a successful or non-successful care pathway.

To our knowledge the present study is the largest and most comprehensive RCT investigating CGA on elderly persons having suffered a hip fracture. There is however need of more research on alternative care pathways [16]. As a second step our research group is now implementing two studies. The first one will focus on potential benefits of a more extensive involvement of and follow-up by the community care system including physiotherapy in the patient's own home to start immediately after discharge from hospital or after returning home from an out-of-hospital rehabilitation facility. This is a case-control study with historic controls from the present study. The second study is a RCT investigating the potential effect of a boost of a 10 weeks intensive physiotherapy program 4 months after the hip fracture.

In conclusion we believe that study design, randomisation procedure and outcome measurements will be of sufficient strength and quality to evaluate important impacts of CGA during the index stay on mobility and other relevant outcomes in hip fracture patients.

## Competing interests

The authors declare that they have no competing interests.

## Authors' contributions

OS initiated the study, has led the work on research design, intervention and implementation of the study protocol and is the primary author of the manuscript. JLH and IS have made substantial contributions to the conception of the study, research design, intervention and implementation of the protocol. PT and KT have made substantial contributions to the implementation of the study protocol, testing and ongoing data collection. SL made important contributions to the research design and conduct of the study. AP has participated in the implementation of the study protocol and is extensively involved in the ongoing data collection. AA, RJ and JM made important contributions to the study protocol. All authors contributed to the writing and review of the manuscript and approved the final version.

## Pre-publication history

The pre-publication history for this paper can be accessed here:

http://www.biomedcentral.com/1471-2318/11/18/prepub
